# Aspects épidémiologiques du suicide à Dakar

**DOI:** 10.11604/pamj.2013.15.10.2221

**Published:** 2013-05-06

**Authors:** Mohamed Maniboliot Soumah, Brice Angwé Eboué, Mor Ndiaye, Mamadou Lamine Sow

**Affiliations:** 1 Service de médecine légale et médecine du travail, Université Cheikh Anta Diop, Dakar, Sénégal; 2 Médecin légiste expert du dommage corporel, médecin du travail, service de santé des armées du Gabon, Hôpital d'instruction des armées Omar Bongo Ondimba, Faculté de médecine et des sciences de la santé, Libreville, Gabon

**Keywords:** Suicide, Afrique, pendaison, Suicide, Africa, hanging

## Abstract

**Introduction:**

Le suicidé est le sujet mort par suicide et le suicidant est la personne ayant fait des tentatives de suicide. L'objectif de cette étude porte sur l'analyse épidémiologique des suicides dans la région de Dakar.

**Méthodes:**

Par une étude rétrospective portant sur les registres du service d'anatomie pathologique de l'Hôpital Aristide Le Dantec, nous rapportons 143 suicides sur 10 ans. Le traitement et l'analyse des données ont été faits sur Epidata version 2.1 b pour la saisie et Epiinfo version 6.04 fr pour l'analyse.

**Résultats:**

A Dakar, les morts par suicide restent peu fréquentes au regard de la mortalité générale. Les hommes se suicident deux fois plus que les femmes et le suicide reste l'apanage de l'adulte jeune dont l'âge se situe entre 21 et 30 ans. Les suicidés résident le plus souvent en zone périurbaine et ils commettent cet acte dans la majorité des cas en période de froid (pendant les mois de janvier, février et mars), plus avant midi et en soirée qu'en après-midi. Aussi 97.2% des suicidés ont utilisé un seul moyen pour se suicider et le suicide complexe (utilisation de plusieurs moyens) a concerné seulement un cas dans notre étude. La pendaison reste le mode le plus utilisé.

**Conclusion:**

Les hommes préfèrent donc des moyens de suicides violents (pendaison, arme à feu et arme blanche) alors que les femmes et les adolescents (tout sexe confondu) utilisent les intoxications. Le recueil des facteurs concourant au suicide permettrait une prévention de ce dernier.

## Introduction

L'ensemble des conduites suicidaires se situe sur un continuum comprenant le suicide abouti, les tentatives de suicides, les projets de suicide, les idéations suicidaires, les comportements autodestructeurs tels que les consommations de toxiques, les sports et la conduite à risque, le jeu pathologique, les pratiques sexuelles à risque et l'automutilation. Le suicidé est le sujet mort par suicide et le suicidant est la personne ayant fait des tentatives de suicide. L'objectif de cette étude porte sur l'analyse épidémiologique des suicides dans la région de Dakar.

## Méthodes

Il s'agit d'une étude rétrospective menée dans le service d'Anatomie pathologique du centre hospitalier national Aristide Le Dantec, qui reçoit les morts de toute la région de Dakar (centre-ville, périurbain, banlieue) et même ceux des autres villes du Sénégal, sur une période de 10 ans (janvier 1996-décembre 2005), durant laquelle nous avons colligé 143 cas de suicide. Nous avons passé en revue de façon systématique tous les rapports d'autopsie et toutes les réquisitions établies par les commissariats de police et les brigades de gendarmerie de la région de Dakar. On retenait donc comme suicide tous les cas classés comme tels par les opérateurs de ces autopsies médico-légales. Le traitement et l'analyse des données ont été faits sur Epidata version 2.1 b pour la saisie et Epiinfo version 6.04 d fr pour l'analyse.

## Résultats

**Figure 1:**
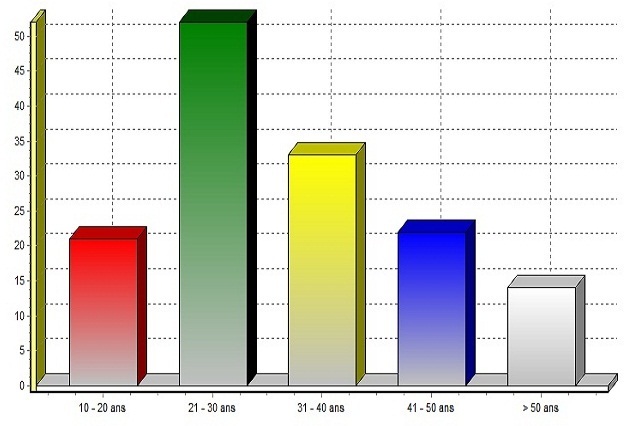
Répartition des suicides selon l'âge

Nous rapportons 143 cas de suicide pendant une période de 10 ans, soit une moyenne de 14.3 cas par an. Sur les 143 suicidés, 99 étaient des hommes (soit 69.2%) et 44 des femmes (soit 30.8%): les hommes se suicident deux fois plus que les femmes. L'âge des suicidés varie de 10 à 75 ans avec une moyenne de 32.7 ans, une variance de 169,662. Dans notre série, 22 ans est la fréquence d'âge la plus relevée (le mode) et 50% des suicidés ont un âge inférieur à 30 ans (la médiane). Il n'y a aucun cas de suicide avant 10 ans. Le suicide est plus fréquent chez l'adulte jeune dont l'âge est compris entre 21 et 30 ans (Figure 1). Le suicide des jeunes entre 10 et 20 ans représente 14.8% des cas de notre série. Les suicidés résident surtout dans la zone périurbaine de Dakar (Tableau 1) qui concentre 50.4% des cas.

**Tableau 1:** Répartition des suicides selon le lieu de résidence

Lieu de résidence	Fréquence	Pourcentage
Dakar plateau	10	7%
Dakar périurbain	72	50.4%
Banlieue	46	32.2%
Hors de Dakar	13	9.1%
Dossiers non renseignés	2	1.3%
**Total**	**143**	**100%**

Dans notre série, 139 suicidés ont utilisé un seul moyen, ce qui représente un pourcentage de 97.2%. Dans un seul cas, le suicidé a utilisé deux moyens pour se suicider soit 0.7%. A l'analyse des procédés utilisés par les suicidés (Tableau 2), nous avons noté une prédominance de la pendaison (44%) suivie par les intoxications le plus souvent de nature médicamenteuse (37%) et les armes à feu (5.6%). Concernant les intoxications, la nivaquine et les insecticides organochlorés (Baygon^®^) restent les principaux produits utilisés. Le suicide par arme blanche est peu fréquent dans notre série (5%), il représente le quatrième moyen de suicide. Il constitue avec le suicide par arme à feu les deux formes généralement retrouvées chez les suicidés de nationalité européenne. Le cas d'association de suicide que nous avons eu concernait une intoxication non réussie couplée à une pendaison.

**Tableau 2:** Répartition des suicides selon le moyen suicidaire

Moyens suicidaires	Fréquence	Pourcentage
Pendaison	63	44%
Intoxication	53	37%
Arme à feu	8	5.6%
Arme blanche	7	5%
Chute de hauteur	6	4.2%
Ecrasement	1	0.7%
Noyade	1	0.7%
Association de moyens	1	0.7%
Dossiers non renseignés	3	2.1%
**Total**	**143**	**100**

La pendaison est prédominante chez les hommes (60 cas sur 63) alors que l'intoxication est un moyen suicidaire de la femme (37 cas sur 53). Les suicides par arme blanche et ceux par arme à feu surviennent exclusivement chez les hommes puisqu'ils représentent respectivement 5.0% (tous les 7 cas) et 5.7% (7 cas sur 8). Les jeunes, dont l'âge se situe entre 10 et 20 ans utilisent plus volontiers l'intoxication comme moyen suicidaire (52.4%) alors que pour les autres tranches d'âge, la pendaison reste le moyen le plus utilisé pour se suicider (52.5%).

Chez les hommes, quelque soit la tranche d'âge considérée, la pendaison reste le moyen le plus utilisé. Chez les femmes par contre, c'est l'intoxication qui est le moyen le plus utilisé avec une prédominance entre 18 et 25 ans. Ces jeunes femmes présentaient un état gravidique au moment du suicide le plus souvent.

## Discussion

Le suicide dans la région de Dakar reste à prédominance masculine. Les adultes jeunes dont l'âge varie entre 21 et 30 ans constituent la population la plus touchée avec un âge moyen de 32.7 ans. La fréquence d'âge la plus élevée est de 22 ans et 50% des suicidés ont moins de 30 ans. La fréquence du suicide dans cette tranche d'âge pourrait s'expliquer par le chômage et le fort taux de déscolarisation dans cette frange de la population. Le suicide est l'une des dix causes principales de mort dans le monde. Selon les estimations de l′OMS, près d′un million de personnes s′est ainsi donné la mort au cours d'une année et 20 à 40 fois plus de sujets ont attenté à leur vie. Un suicide et une tentative de suicide surviennent respectivement toutes les 43 secondes. En Afrique, la fréquence des suicides et des tentatives de suicide est mal connue à cause de la rareté des publications et la quasi inexistence des études dans la plupart des pays. Cependant, dans les pays arabo-musulmans et notamment à Sfax en TUNISIE il représente 10% des décès et vient en deuxième rang des morts violentes derrière les accidents de la circulation routière [1]. En Côte d'Ivoire, les morts par suicide sont peu fréquentes. Elles représentent moins de 1% de l'ensemble des décès et seulement 4.36% des morts violentes [2]. En France, le suicide entraîne près de douze mille (12000) décès annuels, de nombreuses années de vies perdues, de dizaines de milliers d'hospitalisations après tentatives de suicides [3]. Il reste la première cause de mortalité chez les 25-34 ans, et la deuxième chez les 15-24 ans, après les accidents de la circulation. La faiblesse des chiffres relevés en Afrique noire est liée à la forte pénétration et la fréquente pratique religieuse, notamment pour le Sénégal. Ces religions se sont greffées sur une culture où le suicide n'est pas toléré, il n'est pas considéré comme un acte de bravoure comme en Asie ou de désespoir ou déséquilibre en Occident, mais plutôt comme une faiblesse.

Les suicides sont plus fréquents dans certains pays économiquement et intellectuellement développés que dans des contrées où sévit la misère. « La vie trépidante dans les grandes villes modernes ne conduit toutefois pas plus d′individus à se donner la mort que la saine vie à la campagne » [4]. Si l′industrialisation semble parfois provoquer une légère augmentation du taux des suicides, cet accroissement se manifeste surtout parmi la main d′œuvre transplantée. La réduction de la relation humaine à une entité purement économique entraine une désocialisation des travailleurs [5] et l'exemple récent des travailleurs de France Telecom est instructif.

La banlieue Dakaroise est le lieu de survenue de 32.6% des suicides. Cette banlieue est le lieu d'habitation de la main d'œuvre transplantée, résultat de l'exode rural dû à la sécheresse dans les contrées lointaines. Le suicide survient aussi dans la majorité des cas en zone périurbaines, où les familles sont le plus souvent monoparentales et moins nombreuses, le soutien psychoaffectif est par conséquent moins important qu'en banlieue. Cette zone périurbaine représente à peu près la middle class faite d'intellectuels avec des familles à l'occidentale. La fréquence du suicide dans cette frange de la population pourrait s'expliquer par la faible cohésion du tissu social. En effet, Prahbu SL et coll. ont identifié [6] trois facteurs relatifs à la survenue des suicides: la cohésion familiale, l'adhésion familiale et la formation d'une nouvelle famille. Dans la zone périurbaine les deux premiers facteurs sont amoindris tandis que la formation d'une nouvelle famille est un échec pour les populations de la banlieue.

Le suicide se fait généralement à l'aide d'un seul moyen et la pendaison reste le mode le plus utilisé. Il est plus utilisé par les hommes alors que, l'intoxication, qui est le second mode de suicide à Dakar, est plus utilisée par les femmes et notamment les jeunes dont l'âge varie entre 18 et 25 ans. Cela peut s'expliquer par la relative facilité à se procurer le lien ou les produits toxiques contrairement aux armes à feu dont la détention constitue un délit réprimé par la loi en l'absence d'un permis de port d'arme. Le suicide complexe de notre série est du fait de l'enchainement des moyens et non de la combinaison [7–10]. Le suicide par intoxication médicamenteuse est remarquable dans nos pays car il s'agit le plus souvent d'avortement provoqué par la chloroquine: la dose abortive est malheureusement la dose létale. Cela pose un problème bioéthique, l'avortement thérapeutique n'étant prévu dans notre législation que lorsque la vie de la mère est menacée par la poursuite de la grossesse. Quelle solution offrons-nous à ces jeunes filles porteuses de grossesses non désirées, avec un niveau de revenus faible voire inexistant, soumises à la désapprobation de la famille et de la communauté en général, dans des pays où l'adoption n'est pas fréquente ‘ Le suicide résulte du rejet de l'individu par sa famille et ses amis [6].

Pour les autres moyens, si nos constatations sont comparables à celles de Khemakhem [1] et Souguir [11] en Tunisie et Yapo Ette [2] en Côte d'Ivoire, il n'en est pas de même ailleurs. Ainsi, en France et en Europe de façon générale, la pendaison est le deuxième mode de suicide après les armes à feu [12] ou les intoxications. Aux Etats-Unis, les armes à feu restent le premier mode de suicide suivi des intoxications médicamenteuses [13, 14]. Les suicides violents restent l'apanage des hommes [15]. Les femmes utilisent surtout les moyens chimiques.

Par ailleurs, selon les études [4], les suicidés présentent des troubles mentaux dans 90% des cas, et deux diagnostics coexistent dans 70 à 80% des cas. Le suicide est en particulier surreprésenté dans la population des patients souffrant de schizophrénie. La recherche de facteurs favorisants tels que les antécédents médicaux de pathologies psychiatriques et de comportements suicidaires, les addictions éventuelles et les motifs du suicide nous auraient permis de mieux comprendre l'épidémiologie du suicide dans la région Dakaroise.

## Conclusion

Cette étude permet d'attirer l'attention sur la gravité du suicide et confirme la nécessité d'une action préventive pratique et d'une intervention du Médecin Légiste devant toute mort par suicide dès la levée de corps. A Dakar, les morts par suicide restent peu fréquentes au regard de la mortalité générale et leur nombre est probablement sous-estimé du fait des obstacles socio-culturels, religieux, socio-économiques et des dysfonctionnements de la procédure judiciaire.
